# Analysis of Nurses’ and Physicians’ Attitudes, Knowledge, and Perceptions toward Fever in Children: A Systematic Review with Meta-Analysis

**DOI:** 10.3390/ijerph182312444

**Published:** 2021-11-26

**Authors:** Francisco Vicens-Blanes, Rosa Miró-Bonet, Jesús Molina-Mula

**Affiliations:** Department of Nursing and Physiotherapy, Balearic Islands University, Carretera de Valldemossa, km 7.5, 07122 Palma, Spain; Rosa.miro@uib.es (R.M.-B.); jesus.molina@uib.es (J.M.-M.)

**Keywords:** nurses, pediatric, pediatricians, fever, antipyretics

## Abstract

Context: Fever is a common symptom in children that nurses and pediatricians treat. Although it is a common sign in clinical practice, fever instills irrational fears in parents that health professionals share. Objective: To investigate whether doctors’ and nurses’ knowledge, perceptions, and attitudes toward fever influence how this sign is managed. Furthermore, it intends to evaluate whether educational programs increase knowledge and change attitudes and/or perceptions of nurses about children’s fever. Data Sources: A systematic review with meta-analysis was conducted with PRISMA international standards and the Cochrane recommendations. Study selection: Articles examining health professionals’ (doctors and/or nurses) knowledge, perceptions, and/or attitudes toward fever in children and the use of antipyretics were selected for the study. Data extraction: The qualitative analysis was carried out by classifying the articles according to the applied educational programs for nurses related to fever care for children that evaluated different outcomes to determine their efficacies. Results: For the qualitative synthesis, 41 articles were included, and 5 of these were taken in meta-analysis, which measured the effectiveness of educational programs for fever management in nurses. Limitations: All of the included studies generally had a high risk of bias. Conclusion: According to the evidence reviewed, nurses’ and physicians’ perceptions and attitudes regarding fever management in children indicate an overtreatment of this sign. We can give a recommendation grade of D on the use of educational programs to modify attitudes, perceptions, and knowledge about fever in children and improve clinical practice in nurses.

## 1. Introduction

Fever in children is associated with acute childhood illnesses and is a sign that nurses and pediatricians routinely treat [1,2,3,4,5,6,7,8]. According to the authors, although it is a common sign in clinical practice, health professionals share the most common observations with parents, such as brain damage, seizures, and death [1,2,3,4,5,6,7,8].

Fever reduction has shown no effect on morbidity or mortality in children with acute febrile illness. In addition, fever does not lead to tissue injury because it is regulated and self-limited by the hypothalamus [1,2,8,9,10,11,12,13,14,15].

Fever serves several beneficial physiological functions in the host’s defense against infection, including alerting the pathological situation and delaying the growth and reproduction of bacteria and viruses [1,2,3,4,5,7,8,9,10,11,13,14,15,16]. As a result, if fever is not harmful in and of itself, treatment should focus on comfort, with rational use of antipyretic therapies. Moreover, febrile seizures have not been associated with brain damage, and antipyretic treatment has not been reported for their prevention [1,2,3,4,5,7,8,9,10,11,13,14,15,16].

Navarro and de Carlos demonstrated that professionals’ incorrect attitudes toward the febrile child are cultural errors passed down from generation to generation, mainly from fear of febrile seizures and neurological sequelae [17]. The irrationality of these fears is evident when the evidence shows that febrile convulsions do not cause neurological damage and antipyretics do not prevent them, although they are sometimes used for that purpose [3,9,10,13,15,18].

Separating the sign from the underlying condition and understanding the febrile process, according to the authors, allows them to provide the essential care to the child, alerting for signs of serious illness, avoiding dehydration, and ensuring nutrient intake [6,7,11].

In this regard, Razón stated that there is little information about fever in which doctors and nurses are trained and that it causes anxiety in managing the febrile child [1]. In another study, Demir and Sekreter found that 65% of physicians consider this sign harmful, and 85% of pediatricians believe that fever can develop brain damage [19].

A temperature limit for antipyretics administration is a fundamental aspect in reaching a consensus on their usage. According to a study conducted with Australian nurses, using drugs on other aspects such as the child’s discomfort can lead to conflicts with parents and/or peers [20]. Parental influence on antipyretic measures, nursing colleagues, medical professionals, and workload, among other factors, was expressed by Australian nurses [20].

Educational programs have been considered a resource for changing the activities that professionals perform daily in clinical practice. Studies assessing educational programs that were included in this study were aimed to modify the ingrained knowledge, attitudes, and perceptions in nurses and evaluate their efficacy on increased knowledge [21,22], changing attitudes [23], knowledge and attitudes [24], perceptions and attitudes [25] or knowledge, attitudes, and perceptions [26].

The purpose of this systematic review and meta-analysis is to determine how doctors’ and nurses’ knowledge, perceptions, and attitudes toward fever management in children influence their practice. Furthermore, it intends to investigate whether educational programs increase knowledge and change nurses’ attitudes and/or perceptions about children’s fever.

## 2. Materials and Methods

### 2.1. Design

A systematic review with meta-analysis was carried out on doctors’ and nurses’ knowledge, attitudes, and perceptions about fever in children under the age of 14. PRISMA international standards and Cochrane recommendations were followed, and it was registered in PROSPERO on 31 August 2020 (No: CRD42020201362).

### 2.2. Search Strategy

From 15 November 2020 to 15 January 2021, the following databases were used for the bibliographic search: Virtual Health Library, Pubmed, Web of Science, and Cochrane. In addition, EBSCOhost meta-search was conducted with the following selected databases: Psychology and Behavioral Sciences Collection, APA PsycInfo, CINAHL with Full Text, Educational Administration Abstracts, MLA Directory of Periodicals, MLA International Bibliography, APA PsycArticles, E-Journals, eBook Collection (EBSCOhost), Social Work Abstracts, and SocINDEX with Full Text.

The search strategy was developed by truncating the DeCS/MeSH descriptors and a free term with Boolean operators. To avoid losing results, the search formula was: (pediatricians OR nurses, pediatric) and (fever OR Fever Phobia) in addition to a subsequent search with the only free term “fever phobia”.

Subsequently, a directed or snowball search was conducted, which included reviewing the references in the articles as well as those relevant to the study phenomenon that had not appeared due to the included limits. The article selection process was performed in two phases. The titles and abstracts were reviewed first, followed by a full-text reading to determine if they met the inclusion criteria and were of sufficient quality.

### 2.3. Inclusion Criteria

(a) Articles examining health professionals’ (doctors and/or nurses) knowledge, perceptions, and/or attitudes toward fever in children under the age of 14 in hospital and community settings, as well as the use of antipyretics.
(b) Written in English or Spanish.

### 2.4. Exclusion Criteria

(a) Articles that only evaluate fever from a biological perspective.
(b) Research articles on the pharmacological properties of antipyretics.
(c) Articles about fever after vaccination.
(d) Articles assessing the effectiveness of temperature measurement methods.
(e) Articles focusing on parents’ knowledge, perceptions, and/or attitudes toward fever in children.
(f) Articles on the assessment of discomfort in children.
(g) Letters to the editor, comments from experts, and translations of original articles.

### 2.5. Data Collection

The articles were selected in pairs, and any disagreements were resolved by consulting a third researcher. Identification, screening, selection, and inclusion were the four stages of the paper’s selection procedure. All the articles’ titles were scrutinized using the inclusion criteria to eliminate those that were irrelevant. Papers with dubious titles were included in the following phase for in-depth analysis. A summary of each chosen study was reviewed in the third phase of selection to determine doctors’ and nurses’ knowledge, perceptions, and attitudes about fever in children.

An Excel coding sheet was then created for each article: literature reviews, surveys, common practice descriptions, and educational programs. Finally, the meta-analysis included the selected articles containing the evaluation of an educational program for nurses.

The studies’ quality was investigated using the “Critical Appraisal Skills Program (CASPe)” via the online critical reading card tool “FLC 3.0”. According to the criteria applied by this tool, the studies were classified as low, medium, or high quality.

### 2.6. Assessment of the Bias Risk

The bias risk of the articles included in the meta-analysis was evaluated. The authors agreed on the biases of the included research. The risk of bias was assessed using seven domains of the Cochrane Collaboration Tool version 5.1.0 [27], including appropriate sequence generation, allocation concealment, blinding, incomplete outcome measures, selective reporting, and other biases. First, the aspects of the studies related to the aforementioned domains were examined, and then the bias risk was determined. The risk levels were labeled as “low risk”, “high risk”, or “moderate/uncertain risk”. The overall risk of bias for each study was calculated based on the analysis of each domain separately. The rating was identified via the most prevalent risk of bias value in various items of each study.

### 2.7. Analysis and Synthesis

#### 2.7.1. Qualitative Synthesis

The qualitative analysis was carried out to gain a better understanding of the phenomenon under investigation. The variables that influence the care and/or treatment of febrile children by health professionals were investigated. Non-analytical articles, or those that did not establish relationships between variables, were listed in this category of analysis. All of the articles could not be included in the meta-analysis due to their heterogeneity and non-analytical conditions.

#### 2.7.2. Quantitative Synthesis

After that, studies with an analytical nature were considered for the meta-analysis. The articles evaluating educational programs for nurses that had at least two statistical measures of the variables were extracted. The Meta-Essentials Excel tool was used to create the meta-analysis. The articles’ analyses were divided into three groups based on the variables: those that evaluated knowledge, and those that investigated the attitudes and/or perceptions. If an article examined more than one variable, it might be classified as belonging to more than one category.

All the articles included in the meta-analysis shared the relationship of the variables through the mean and standard deviation (SD). Therefore, these two measures were chosen to compare the results of the various articles and draw conclusions.

The mean and SD of each variable were extracted from the pre-test and post-test results of the selected studies, depending on whether their intervention included an experimental and control group or only an experimental group. The standardized mean difference (SMD) at 95% confidence intervals (CI) was calculated by dividing the mean difference between the experimental and control groups by the SD of both groups. In each of Cohen’s studies, SMDs in the means were weighted by the inverse of their variance to obtain the pooled index of the magnitude of the effect. A random-effects model was selected due to the high heterogeneity of the studies.

The differences between the averages of the pre-test and post-test of experimental and control groups were calculated to determine the size effect of variables. Subsequently, the difference between the mean of the experimental and control group’s pre-tests was assessed. Accordingly, the size effect could be obtained by adding this difference to the experimental group mean difference based on the relation proposed by Cohen.

The magnitude effect of the involved variables was calculated for one of the studies that did not have a control group but had a pre-test and post-test of a single group. This was obtained by dividing the difference in the standardized means of the pre-test and post-test proposed by Cohen via the post-test SD. The magnitude effect was calculated using Rosenthal’s r because in a study lacking the mean and SD data (Considine & Brennan, 2007). The Z value was extracted by taking the square root of U Mann Whitney’s with N, which has the same properties as Cohen’s d.

Heterogeneity was assessed using the inferential Q test proposed by Cochrane and the I2 index of heterogeneity with its 95% CI. When I2 was more than 50%, heterogeneity was considered as high. The size effect was interpreted using the following thresholds: 0.2-small, 0.5-medium, and 0.8-large. The *p*-value of 0.05 was also used to determine statistical significance.

## 3. Results

### 3.1. Search Results

**Figure 1:**
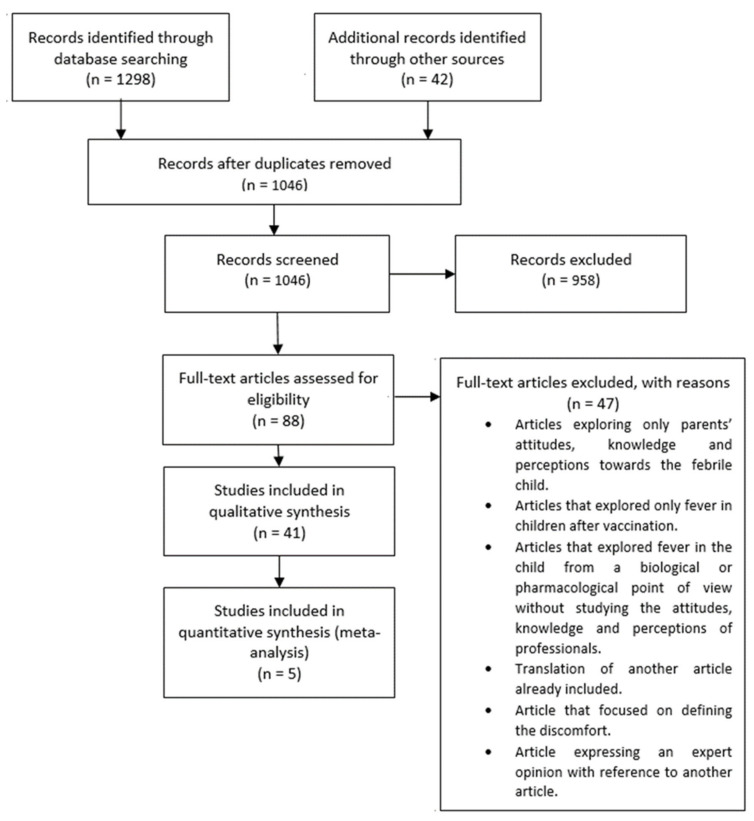
PRISMA flowchart. From: Moher D, Liberati A, Tetzlaff J, Altman DG, The PRISMA Group (2009). Preferred Reporting Items for Systematic Reviews and Meta-Analyses: The PRISMA Statement. PLoS Med 6(6): e1000097. doi:10.1371/journal.pmed1000097.

The search was completed in January 2021, with 1298 articles discovered in databases and 42 articles found using the “snowball” technique. After removing duplicates, there were 1046 articles left. 88 of the aforementioned cases were evaluated in full text for inclusion in the study. In addition, 47 papers were excluded for the following reasons: Concentration on parental knowledge, perceptions, and attitudes toward fever in children, focus on fever after vaccination, studying fever solely from a biological or pharmacological standpoint, defining malaise, and stating expert opinions as well as translations. Finally, 46 articles were derived. Forty-one were included in the qualitative synthesis, while the quantitative synthesis comprised 5 cases. This information is represented in Figure 1: PRISMA flowchart.

The included articles were then used to create two tables. Table 1 lists the articles included in the qualitative synthesis. This table provides the following information for each paper: design, data collection method, objectives, location and date, population and sample, results, conclusions, quality, and level of evidence. The second table (Table 2) summarizes articles that evaluated educational programs and could be used in the quantitative analysis. Therefore, the following sections were added to Table 2: Intervention and comparison, the number of participants in intervention and control groups, the measurement instrument applied, and the variables analyzed.

### 3.2. Description of the Included Studies

There are 18 bibliographic reviews (44%), 1 systematic review (2.4%), 21 studies with quantitative methodology (51.3%), and 1 qualitative research (2.4%) among the 41 articles included.

Among the qualitatively analyzed articles that used quantitative methods, descriptive ones using self-administered questionnaires were included (*n* = 12) [19,28,29,30,31,32,33,34,35,36,37,38], one of which was an audit of antipyretics administration records in hospitalized children [39].

Studies collected data on doctors’ practices (*n* = 9) [19,28,31,32,33,34,37,38,40], nurses’ practices (*n* = 4) [29,30,36,39], or both (*n* = 2) [35,41].

The descriptive studies included 472 nurses; 83 stated that they had training in pediatrics, but the majority referred to pediatric nurses; however, it was not specified whether the training was a regulated or official postgraduate course. The total number of doctors considered was 4651, with 4343 being pediatricians and 20 being resident doctors. Within the quantitative studies, quasi-experimental evaluating educational program effectiveness stood out (*n* = 6) [21,22,23,24,25,26]. There were 293 pediatric hospital nurses in total. The meta-analysis included five studies, one of which was excluded due to a lack of statistical data.

The meta-analysis was conducted with five studies that included samples from 59 Korean nurses [26], 126 Turkish nurses [21], 31 Australian nurses [22], and two other studies that did not specify the sample of Australian nurses used [20,24,25]. According to the bias analysis, none of the studies used adequate randomization for sample selection. Gender was only specified in two of the quantitative studies included in the investigation.

The average age of the participants was 31.51 years, with a standard deviation of 7.5 years [21,22,24,25,26]. In terms of participant distribution and design, 3 presented an experimental and control group, administrating pre-tests and post-tests in both groups [26], or including a “latency test” carried out 4 months after the educational intervention [24,25]. There were no procedures for group randomization used. The other three studies only presented an intervention category with the evaluation of a pre-test and a post-test in the same group [21,22]. Finally, a qualitative methodology based on focus groups in an Australian hospital with 15 nurses was included [20].

### 3.3. Variables Measured in the Educational Programs

#### 3.3.1. Qualitative Analysis

The qualitative analysis was carried out by classifying the articles by educational programs for nurses that evaluated different outcomes to measure the efficacy of the studied program related to fever care for children, which would later be included in the quantitative meta-analysis. These classes were attitudes, knowledge, and perceptions.

As a result, the research was classified according to the variable they measured. When the evaluation of the education programs probed nurses’ understanding of fever physiology, fever management, and antipyretic drugs or measures, the results were categorized as knowledge. The attitude class contained tests that assessed changes in the professionals’ clinical practice regarding the management of a febrile child, performance against febrile convulsions, and the health education they would provide to parents following the training. The perceptions category included results relating to how parents, other nursing colleagues, and physicians influence the care of a febrile child, and how much control they have over the management of the febrile child.

The analyzed studies employed various educational methods and attempted to adjust these variables as well as different evaluation methods to determine whether these programs are effective. A Korean study compared a “blended learning program” (which combines traditional classes with online learning) to “face-to-face lessons” (Traditional classes). Based on the findings, there was no difference in effectiveness between the two methods, but the intervention group had higher satisfaction in both methodologies through pre-test and post-test [26]. Another study measured the increase in knowledge between the pre-test and the post-test for those who were given a “training booklet”. The authors specified that those who were provided the “training booklet,” had a slight increase in knowledge, but when each professional read this information on their own, it was impossible to control whether this reading was done correctly [21]. Two Australian studies compared the prior and subsequent knowledge of a group of nurses after receiving two tutorials. Only 45.2% of the participants completed the tutorials, and the rest of the professionals were given the information in writing prior to the evaluation [22,23]. Two other studies with the same sample compared the pre-test, post-test, and latency test of nurses who had participated in a peer education program to those who continued with their usual practice but did not specify which sample was the control group [24,25].

The studies were all performed in hospitals. Because of staff mobility, which resulted in the loss of professionals who changed units or terminated their contracts [24,25], the sampling method used was convenience [21,22,23], selecting nurses from two children’s hospitals [26], or choosing entire units. Although there were differences in nurse specialization, work experience, and unit category, no prior selection criteria were established. It should be noted that the majority of the sample’s losses occurred during the follow-up and evaluation of the educational program. Therefore, the studies included had a high risk of bias in participant selection.

In terms of questionnaires, all educational programs used a structured self-administered questionnaire in their tests. Four of them applied or adapted the “fever management survey” developed by Walsh et al. in 2005, which is divided into three other questionnaires: fever management knowledge (FMK), fever management attitudes (FMA), and fever management practices (FMP) [23,24,25,26]. The study authors created and validated one of them to assess knowledge [22] or used an unvalidated questionnaire to evaluate knowledge [21].

#### 3.3.2. Quantitative Analysis and Meta-Analysis

**Figure 2:**
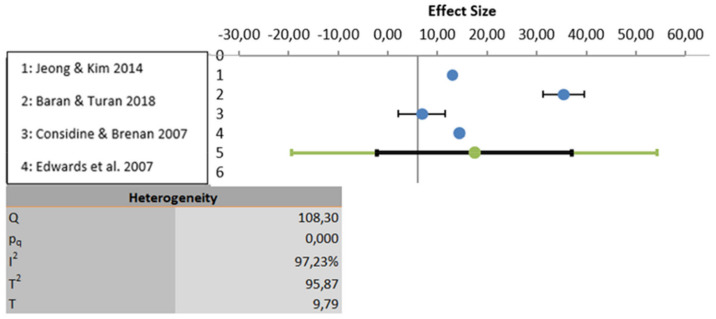
Data analyzed for the variable knowledge.

**Figure 3:**
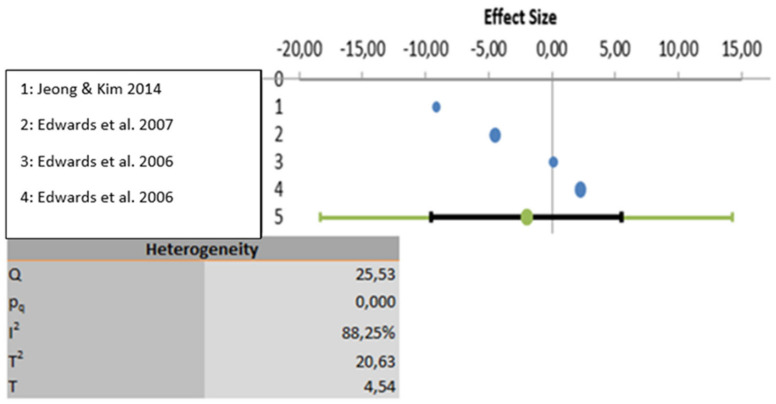
Data analyzed for the variable attitudes.

**Figure 4:**
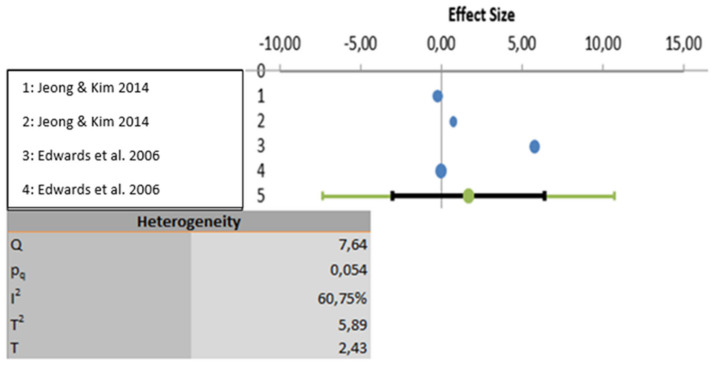
Data analyzed for the variable perceptions.

The meta-analysis was carried out based on five studies that evaluated educational programs [21,22,24,25,26]. One study was excluded because the results were not properly analyzed using statistical data [23]. All three variables show a high degree of heterogeneity. In the first-place knowledge is the variable with the highest heterogeneity (I2: 97.23%), followed by the variable attitudes (I2: 88.25%) and lastly the variable perceptions show the least heterogeneity (I2: 60.75%). Considering statistical significance, the knowledge and attitude variables show statistically significant results (*p* < 0.001) but the perceptions variable does not present statically significant result (*p* > 0.005). These analyses are depicted graphically in Figure 2, Figure 3 and Figure 4.

### 3.4. Risk of Bias

**Figure 5:**
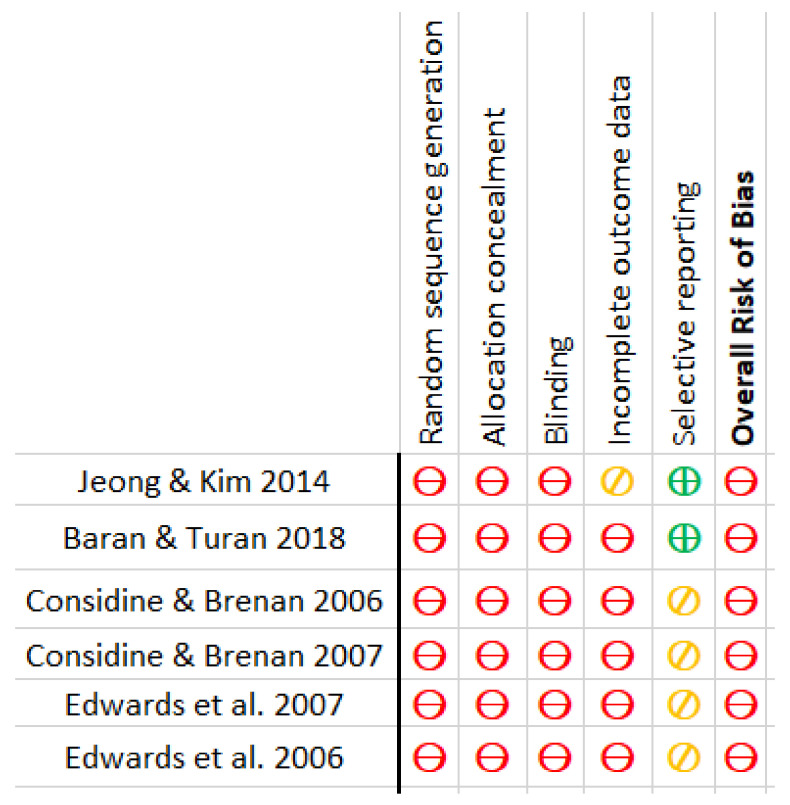
Risk of Bias.

**Figure 6:**
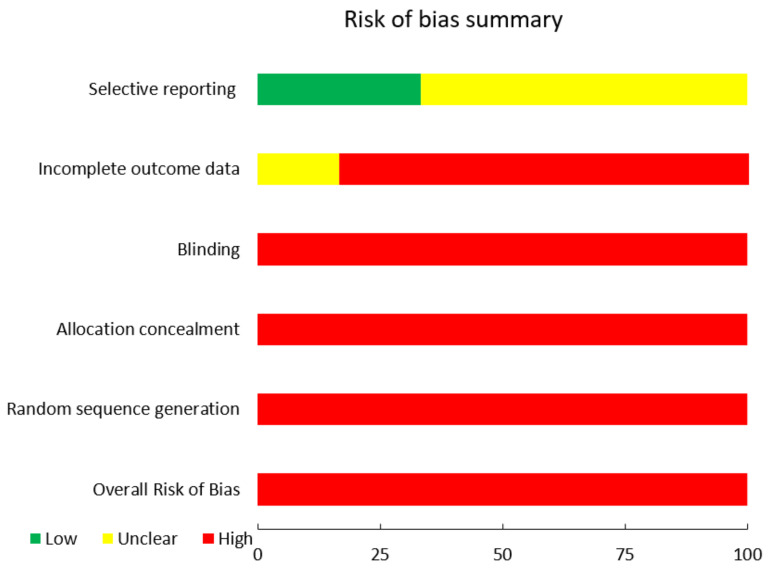
Risk of Bias Summary.

In general, all of the included studies had a high risk of bias. In addition, all were at high risk of insufficient sequence generation, allocation concealment, and blinding [21,22,23,24,25,26]. With the exception of one, all had a high risk of bias due to incomplete outcome measures [26]. Two of the reports chosen had a low risk of bias [21,26], while the others had a moderate risk level [22,23,24,25]. The risk of bias in the included articles is graphically depicted in Figure 5 and Figure 6.

## 4. Discussion

This systematic review included 41 studies to determine whether the knowledge, perceptions, and attitudes toward fever by doctors and nurses who work with children affect antipyretic measures. In addition, we aimed to assess if the educational programs included in the meta-analysis could lead to changes in the usual clinical practice of nursing care of the febrile child.

As Razón points out, lack of knowledge and understanding of this process leads to the use of aggressive treatments to achieve normothermia, such as combination antipyretic therapy [1]

Nurses and doctors agreed that fever might be beneficial, but they were concerned about the long-term consequences. For example, 50% of nurses in Ireland supposed that fever has beneficial effects on the immune system, and 84.9% reflected that regular paracetamol usage could disguise symptoms, but that fever should be treated rapidly to avoid febrile seizures [29].

The research demonstrated that a temperature limit, rather than discomfort, was the most important criterion for providing antipyretics. According to Radhi’s study, most physicians believe that antipyretic medication is intended to reduce fever symptoms; therefore, doctors tend to prescribe antipyretics for every child with this sign. As a result, it could be given to a child who is depressed as well as a playing child [5,9].

In one study, parents were advised to use antipyretics whenever the temperature rose above 38.3 °C [28]. Another study reported the temperature limits used by Argentine doctors to administer antipyretics, when 49% managed it at 38 °C or lower [37]. In a Spanish study, only three doctors advocated the general condition as a criterion for delivering antipyretics. Meanwhile, 67.8% of primary care pediatricians and 66.7% of hospital pediatricians recommended antipyretics when the temperature reached 38 °C [38]. 

According to an audit conducted in a hospital of the same nationality, 45% of antipyretics were given at temperatures below 38.3 °C. Although the authors of a recent study clarified that these medications could also be used for purposes other than temperature control, such as pain relief or discomfort, the reasons for their use were not explicitly stated in the reviewed article [39].

According to the pediatricians who participated in the Martins & Abecasis study, fever is a healthy physiological process for the immune system, and the child’s health should be considered during treatment due to the discomfort it may cause. However, antipyretics are still recommended by 78.1% of family doctors and 81.4% of pediatricians [35]. 

A mixture of antipyretics was shown to be effective in lowering body temperature. However, its safety, efficacy in improving the child’s comfort, and other clinical outcomes are still questioned [2,3,4,8,11,15]. Even so, only 15% thought the child’s discomfort as the first symptom [41]. Similarly, 76.1% of a Spanish pediatrician sample maintained this practice, with the caveat that it should only be used in exceptional cases [38].

Physical treatments such as applying the cold compresses or removing the child’s clothing would counter fever treatment [2,4,5,8,11,16]. Other examples using these strategies in pediatricians could be found in the Lava et al. research, where only 7% of pediatricians prescribe antipyretic therapy as an alternative. Whereas 65% recommended physical temperature lowering strategies [31].

The usage or recommendation of various temperature reduction methods by doctors and nurses has also been examined through descriptive research, and the practice may have changed over time. As an example, studies attended by Chiappini et al. in 2009, 2012, and 2015 clarified that the percentage of pediatricians using alternative antipyretic therapy or suggesting it to reduce the incidence of febrile seizures has decreased from 27% to 12.2%. There has also been a reduction in the recommendation of physical measures from 65% to 52%. Although, the recommendation of thinking about the child’s discomfort rather than a temperature was declined from 45.3 to 38.2% [33,34,40].

Notably the present review has focused on the knowledge, attitudes, and perceptions of professionals about fever in children, however several studies have shown that how professionals treat fever influences parents.

In this regard, it is worth noting that most authors seem to agree that the aggressive fever management by professionals promotes parental fear of this sign and their desire to achieve normothermia in their children [2,3,10,14,17]. This results in a rebound effect in which the parents’ anxiety influences the professionals, who seek to quickly resolve fever to satisfy them and reduce their anxiety [1,5,7]. When asked about this, pediatricians denied reducing fever to calm parents (81% and 63%) [31,32]. Moreover, the nurses did mention that parents were pressuring them to provide their children antipyretics [20]. 

Consequently, researchers have developed actions to change nurses’ attitudes, perceptions, and/or knowledge in pediatric practice by measuring the efficacy of various educational methods.

Edwards’s examination demonstrated that peer education could increase general knowledge about fever but did not significantly improve knowledge about antipyretics. In terms of attitudes, they reported a significant improvement [24]. There were no differences in attitudes toward the efficacy of antipyretics between groups. In contrast, in the experimental group, the perception of control increased, and the intention to use antipyretics decreased [25].

Jeong and Kim compared a hybrid online and face-to-face approach versus a traditional method. Based on the findings, both the control and intervention groups significantly improved their knowledge of fever, attitudes, and intentions to use antipyretics. Nevertheless, regulatory influences and the perception of control did not change significantly. As a result, while the type of education did not improve the traditional method, it resulted in a higher level of satisfaction [26]. In other cases, using a training book slightly increased their knowledge [21]. Another study measured face-to-face tutorials and concluded that they improve knowledge and clinical practice [22,23]. In general, meta-analysis revealed that educational methods cause a statistically significant change in knowledge and attitudes, as opposed to perceptions which did not show a statistically significant change.

There are some limitations to this review. Due to language limitations, relevant studies might have been left out. In addition, the use of unvalidated questionnaires in articles may restrict the validity of the results. Furthermore, the study phenomenon, i.e., attitudes, knowledge, and perceptions, are variables that are difficult to quantify.

## 5. Conclusions

The attitudes, knowledge, and perceptions of health professionals that lead to overtreatment and overestimation of fever in children have received little attention. According to the reviewed literature, the way professionals understand fever and how they respond to it may result in fever management in children based on overtreatment and overestimation of fever and its complications, reflecting a possible irrational fear of this sign. On the one hand, most studies are descriptive and do not investigate these issues analytically, making it difficult to draw conclusions with a high level of evidence. Existing studies that evaluate educational programs, on the other hand, are an intriguing approach to this phenomenon, as they attempt to change knowledge, perceptions, and attitudes to modify daily clinical practice. Meanwhile, they still present a high risk of bias, and their efficacy cannot be affirmed. A qualitative study could delve deeper into the phenomenon, determining the reasons and acting based on them. The majority of the included studies, according to the SIGN scale “Scottish Intercollegiate Guidelines Network”, have a descriptive evidence level of 3 or a quasi-experimental level of 2. As a result, we can give a grade of recommendation D on the use of educational programs for the modification of attitudes, perceptions, and knowledge about fever in children and the improvement of clinical practice in nurses. Hence, the interventions evaluated cannot be recommended or discouraged.

## Figures and Tables

**Table 1:** Quality of studies measured by “Critical Appraisal Skills Programme España” (CASPe) *. Levels of evidence used: “Scottish Intercollegiate Guidelines Network” SIGN (for studies with quantitative methodology) **; Gálvez Toro method (for studies with qualitative methodology) ***.

First Author & Year	Design	Data Collection	Objectives	Location and Date of Execution	Population and Sample	Results	Conclusions	Quality of the Study (CASPe *)	Levels of Evidence (SIGN **) (Gálvez Toro ***)
El Khoury et al. 2010	Descriptive	Questionnaire	Understanding the perception and management of fever in children up to 6 months of age by pediatricians in the United States.	United States. December 2008.	**Population**: 35,600 pediatricians from the “Physicians’ desk reference” **Sample**: 401 American pediatricians subscribed to the “Physicians’ desk reference”.	On average, in children divided into the three age groups (0–2 months, 2–4 months and 4–6 months), respondents indicated that they considered temperatures between 37.8–38.1 °C to be mild, 38.3–38.7 °C to be moderate, 38.8–39.5 °C to be severe and 39.5–40.3 °C to be extremely severe. In general, respondents indicated that they would recommend the use of antipyretic drugs at temperatures of around 38.3 °C.	Pediatricians in the United States are more concerned about general fever than post-vaccination fever. The management and definitions of fever severity by sample pediatricians depend on the age of the child. Recommendations for fever control depended on the level of fever, the age of the child, the timing of vaccination, and the time of day the fever was reported.	MEDIUM	3
Lava et al. 2012	Descriptive	Questionnaire	The aim of this study was to describe the treatment of children with fever by pediatricians in Switzerland.	Switzerland. From June 2010 to March 2011.	**Population**: 900 pediatricians (72% of certified pediatricians in Switzerland). **Sample**: 322 Swiss pediatricians.	2/3 of the participants indicated that sometimes or often a fever that does not respond to antipyretics suggests an underlying bacterial infection. In these cases, half of the pediatricians add a second drug to the existing regimen, about a quarter continue the original treatment, and the remaining quarter replace the initial drug with another. Almost all respondents (92%) indicated that they believed that the exaggerated fear of fever was widespread. However, 81% of respondents indicated that they do not lower the temperature threshold solely to reassure parents.	The results of the Swiss national survey on fever management among pediatricians suggest that the child’s overall appearance and comfort are now recognized as the most important factors in initiating antipyretic treatment. However, the survey also suggests that there is often a gap between what scientific evidence has found to be the most effective intervention and clinical practice. The data could be used as a basis for developing practical guidelines for the treatment of children with fever based on available scientific evidence, while considering current practices of pediatricians, which are influenced in a several irrational factors.	MEDIUM	3
Bettinelli et al. 2013	Descriptive	Questionnaire	Investigate whether hospital pediatricians, community pediatricians and paediatric residents differ in their day-to-day clinical practice with respect to compliance with available guidelines on fever control.	Italy. June to September 2012.	**Population**: 118 pediatricians from the northern area of Lombardy were invited to participate (41 hospital pediatricians, 48 community pediatricians and 29 paediatric residents). **Sample**: 79 pediatricians: 29 hospital, 30 community and 20 residents.	The management of a child who is comfortable and whose fever does not respond to the first antipyretic differs between groups: Paediatric residents replace the first drug with another antipyretic (50%) or, more rarely, add a second drug to the existing regimen (20%) more often than community pediatricians (20% and 3%) and hospital pediatricians (10% and 7%). Physical antipyretic methods are used in all groups by at least 59% of participants, with no significant differences between groups. Similarly, in all groups, 86% of participants believe that it is sometimes or often possible to educate and reassure families about the fear of fever.	This exploratory study demonstrates limited disagreement among paediatric residents, community pediatricians and hospital pediatricians regarding the management of symptomatic fever.	MEDIUM	3
Chiappini et al. 2012	Descriptive	Questionnaire	Investigate medical and parental knowledge and management of fever in preschool children.	Italy. November 2009, June 2010.	**Population**: 648 pediatricians who attended the 14th National Congress of Practice Paediatrics, held in Florence in November 2009 All parents of children aged 0–6 attending 12 public day care centres, all located in the same location (Lastra a Signa, Florence, Italy) were invited to participate in the study. **Sample**: 4338 parents and 480 pediatricians.	The temperature that pediatricians consider as fever was higher than 37.0 °C for 14.3%; 37.5 °C for 32.7%; 38.0 °C for 41.2%. 69% of pediatricians stated that they would give antipyretics for temperatures >38.5 °C; 17.7% above 38.0 °C and 11.6% above 39.0 °C. 65% of pediatricians said they would recommend physical methods, such as the use of sponges or ice packs, to reduce fever only if the temperature did not drop after the antipyretic drug. 13% reported suggesting the use of physical methods in association with antipyretics. Contrary to the recommendations in the guidelines, the preventive use of paracetamol or ibuprofen for the prevention of febrile seizures in febrile children was recommended in 60.6% of pediatricians.	Parents consider pediatricians as their main source of information, and this is also demonstrated by the consistency between the responses of the two groups. Some of the behaviours identified (widespread use of suppositories, alternating use of antipyretics, use of spoons and teaspoons to dose antipyretics) expose children to the risk of overdose. Educational programmes to educate pediatricians can be an effective action to change parents’ understanding and management of fever.	MEDIUM	3
Chiappini et al. 2013	Descriptive	Questionnaire	To evaluate the impact of the publication of the IFG “Italian fever guidelines” on the knowledge and behaviour of a sample of Italian pediatricians, by administering the same questionnaire before and three years after its publication.	Italy. November 2009 to June 2010.	**Population**: The questionnaire was first administered to all the pediatricians who attended the “14th Italian National Congress of Practice Paediatrics” in Florence in November 2009. The same questionnaire was administered to the pediatricians who attended the “12th National Congress of Italian Society of Paediatric Infectious Diseases”, held in Florence in 2012. **Sample**: 480 pediatricians in 2009 and 300 pediatricians in 2012.	In both surveys, most pediatricians recommended the use of physical methods if the fever persisted over time. In 2009 only 11% of the pediatricians, correctly, clarified that there was no temperature cut-off to initiate the use of antipyretics, but that it depends on the patient’s discomfort; while in 2012 a higher percentage of pediatricians, 45.3%, declared it. Contrary to the GIF recommendations, in 2009 27.0% of the participants declared to recommend the alternative use of ibuprofen and paracetamol. This proportion decreased to 11.3% in 2012.	The findings underline the importance of disseminating the IFG to improve pediatricians’ knowledge of fever. Some misbehaviours, such as the alternate use of antipyretics and their rectal administration in the absence of vomiting, need to be further discouraged. An additional strategy may be needed to disseminate the IFG through other channels and to remove possible barriers to adherence to the IFG.	MEDIUM	3
Chiappini et al. 2018	Descriptive	Questionnaire	To know the management of fever by Italian pediatricians 6 years after the publication of the IFG “Italian fever guidelines” and to compare it with the questionnaire carried out 3 years after its publication.	Italy. 2009–2012.	**Population**: Pediatricians who attended the “National Congress of Practice Paediatrics, held in Florence in November 2015 and pediatricians who attended the “12th National Congress of the Italian Society of Paediatric Infectious Diseases” held in Florence in 2012. **Sample**: 300 in 2012 and 562 in 2015.	48.0% of pediatricians never recommended wet dressings, ice packs and other physical methods (not recommended by the IFG) in 2015, a significant increase from the results reported in the 2012 survey (36.4%). The use of antipyretics based on the presence of discomfort, and not for a specific cut in body temperature, was recommended by only 38.2% of pediatricians.	The article highlights improvements in the management of the febrile child in Italy, but also detects the persistence of some incorrect habits. Several key messages from the IFG need to be further highlighted. In particular, the recommendation concerning the use of antipyretics according to the child’s discomfort seems to be adopted only by a minority of pediatricians. Similarly, recent literature reports suggest that improvements in educational interventions are needed in many European countries. Our results may be helpful in guiding educational interventions and compliance with IFG recommended practices. More studies are needed to understand the “weak points” of communication between Scientific Societies and pediatricians, as well as between pediatricians and carers.	MEDIUM	3
Martins & Abecasis 2016	Descriptive	Questionnaire	To assess the knowledge of professionals and parents about fever in children.	Portugal. December 2013 to April 2014.	**Population**: Accompanying parents of children visiting the emergency room in two hospitals in Lisbon. The questionnaire was available online for health professionals; it was sent by e-mail to all nurses working in the paediatric wards of both hospitals and to all pediatricians and family doctors registered with the Portuguese Paediatric Society and the Association of General and Family Medicine respectively. **Sample**: 49 nurses, 228 family doctors, 291 pediatricians and 256 parents.	Most professionals agreed that “Fever is a benign physiological mechanism that contributes to the function of the immune system. On a scale of one to five, pediatricians assigned an average score of 4.59, family doctors 4.43 and nurses 4.27. The average temperature treated by nurses was 38.09 °C by family doctors was 38.07 °C and by pediatricians was 38.14 °C. The attitude towards a feverish child differs between professional groups. Most nurses (67.3%) agreed that “a child with fever should be treated regardless of his/her general appearance and symptoms”, but most doctors did not agree, namely 61.8% of family doctors and 63.6% of pediatricians. Most nurses (90.9%) also thought that a child should be woken up to take antipyretics. Only 44.7% of family doctors and 41.9% of pediatricians agreed with this. A history of febrile seizures resulted in higher scores for nurses and family doctors, while pediatricians considered this factor to be significantly less important. Alternating antipyretics was a common practice among health professionals: 100% of nurses, 78.1% of family doctors and 81.4% of pediatricians recommended it.	The attitudes and beliefs of parents and nurses were found to show fear of fever and concern about its possible consequences. Family doctors shared some of these concerns. Despite having different concepts and opinions, pediatricians did not always have a different approach. Educational interventions are needed for all groups to avoid the perpetuation of fever phobia.	MEDIUM	3
Mayoral et al. 2000	Descriptive	Questionnaire	Identify fever control strategies, their rationale, and assess the frequency of alternating acetaminophen and ibuprofen.	United States of America. February to October 1998.	US pediatricians, paediatric nurses and family doctors recruited at clinical meetings. **Sample**: 161 paediatric professionals (68% general pediatricians in private practice. 17% hospital pediatricians. 15% family doctors, specialist pediatricians and paediatric nurses).	21 participants (13%) used discomfort as the main indication for the use of antipyretics regardless of temperature. When asked if they advised parents to alternate between paracetamol and ibuprofen, 80 out of 161 participants answered in the affirmative (50%).	The survey showed that alternating antipyretics is a common practice among pediatricians. Alternating antipyretics could be the result of fear of fever. The likelihood of alternating antipyretics increased as the number of years in practice decreased. This could mean that, less experienced pediatricians are more likely to succumb to the phobia of parental fever, becoming part of the problem, while more experienced pediatricians are more likely to be unaffected. Alternating acetaminophen and ibuprofen can be confusing for caregivers, which can result in an incorrect dose of either product; this can lead to a double dose increasing the risk of toxicity.	LOW	3
Walsh et al. 2006	Descriptive	Questionnaire	To examine the influence of the level of practice, additional education and paediatric experience on nurses’ knowledge and beliefs about fever and its treatment.	Australia. Does not specify date of completion.	**Population**: Level 1 and level 2 nurses working on the floors of an Australian metropolitan children’s hospital. **Sample**: 51 paediatric nurses.	General knowledge was unsatisfactory (average 12.4 out of 20) Nurses’ knowledge of antipyretics and their use in treating fever was also low (5 questions, Average 2.84). Level 2 nurses had more general knowledge. Nurses with one to four years of paediatric experience had more general knowledge and knowledge of the physiology of fever than those with less than one year of experience. Nurses who had undertaken additional paediatric education had much better knowledge of the physiology of fever. General beliefs about fever and fever control were positive; for example, 60% believed that fever was not necessarily related to the severity of the illness and 75% reported that children with cardiac and/or respiratory disorders were “at risk” for fever. However, several negative beliefs were identified that would significantly affect practice. More than half of the nurses (57%) believed that their peers were phobic of fever and that fevers below 41 °C could be harmful to children (61%). Some determined the need for antipyretic administration by temperature measurement alone (39%) and reduced all temperatures of 38.3 °C and above (39%), even when the child was asleep (37%). Most believed that fever needed to be treated aggressively in children with a history of febrile seizures (85%).	This study identified that level 2 nurses and nurses with one to four years of paediatric experience knew the most about fever and its control. However, this knowledge did not positively influence their beliefs; their beliefs were like those of novice paediatric nurses. It is essential that learning “on the job” is evidence-based. Programmes should focus on beliefs and knowledge, as higher levels of knowledge in fever management do not positively influence nurses’ beliefs.	MEDIUM	3
Melamud et al. 2008	Descriptive	Questionnaire	To know how pediatricians handle fever; considering: the frequency with which they use antipyretics, the alternation of different drugs, and the use of non-pharmacological measures.	Argentina. July 2005.	**Population**: Doctors registered on the Intramed website. **Sample**: 1599 pediatricians.	49% indicated an antipyretic from 38 ºC, 16% from 37.8 ºC and 12.5% from 37.7 ºC. 15.5% began to administrate antipyretics at 38.5 ºC. 96% used physical methods and 95% combined them with antipyretics.	t could be observed that the antipyretic of choice did not vary significantly in relation to the years of professional practice and that the factor statistically linked to the alternation of antipyretics was to have less than 20 years in the profession. From the multifactorial analysis we have been able to establish that those who most combine means The use of antipyretics is based on the patient’s clinical experience and those who alternate less, on scientific publications.	LOW	3
García Puga et al. 2012	Descriptive	Questionnaire	To evaluate the advice given to parents about fever and to know the estimated incidence of fever without outbreak in consultation, the accessibility of complementary examinations and the application of a protocol.	Spain. May to September 2009.	**Population**: All medical professionals in the centres included. Primary care pediatricians from two health areas, pediatricians and residents from two hospitals. **Sample**: 109 primary care pediatricians (PAP), hospital pediatricians (PH) and paediatric residents (R).	67.8% of primary care pediatricians, 66.7% of hospital pediatricians and 91.7% of residents recommend starting the administration of antipyretics, depending on the temperature of 38 °C in the armpit. Primary care pediatricians are divided between those who administer antipyretics from 38 °C (52.9%) and those who administer them from 38.5 °C (38.2%). There are no significant differences between professionals regarding the indication to treat with antipyretics according to the degree of temperature, in which part of the body and with which thermometer to take it.	There seems to be little agreement on how to transmit a definition of fever to parents and how to apply the protocol, but not on the use of the instrument for measuring temperature and treating fever, both physical and pharmacological. There is a wide variability in what professionals consider as Fever Without Focus diagnosis, being considered higher among those working at hospital level. Accessibility to complementary examinations by primary care professionals is very low, which does not facilitate the application of the protocol. Although there is a good knowledge of the process there is a low practice of it.	MEDIUM	3
Demir & Sekreter 2012	Descriptive	Questionnaire	Identify the knowledge, attitudes and misconceptions of primary care physicians regarding fever in children	Turkey. April to May 2010.	**Population**: Primary care physicians in a Turkish province. **Sample**: 80 primary care physicians.	Approximately two-thirds of physicians (73.8%) reported that they recommend an antipyretic agent to all children under 5 years of age with fever. Only 26.2% of the doctors considered signs and symptoms other than fever (malaise, irritability, prolonged crying, signs of infection) when prescribing the antipyretic. Most doctors (90%) indicated that febrile seizures can cause brain damage. More than half (65.0%) of the doctors said that fever is harmful to the child and 70.7% of them reported that a body temperature above 38 °C should be treated, whatever the underlying pathology. Many (76%) believed that the main reason for the use of antipyretics is to prevent febrile seizures and 87.5% indicated that physical methods (bathing) should be recommended to reduce fever. the body with alcohol. 78.7% agreed that paracetamol and ibuprofen can be used in alternative treatment.	The data suggest the need to implement educational programmes and use guidelines for the appropriate management of the child with fever. There are misconceptions about the management and complications of fever. Conflicting results on fever in the literature also confirm these misconceptions.	MEDIUM	3
Greensmith 2012	Descriptive	Questionnaire	Describe the knowledge and attitudes towards fever management of nurses in a children’s hospital.	Ireland. 2012.	**Population**: All the nurses who worked at the Irish Children’s Hospital. **Sample**: 370 nurses.	Of the 20 knowledge questions, 50.9% were answered correctly. 60% agreed that temperature was not related to the severity of the illness, and 66% correctly agreed that children with pre-existing cardiac and respiratory disorders had a lower tolerance for fever. However, only 50% believed that fever had beneficial effects on children. 84.9% of the nurses correctly agreed that regular administration of paracetamol could mask a fever indicative of progressive infection. Most (73.9%) would wake a sleeping child with a temperature above 38.3 °C to administer an antipyretic. A large majority of nurses (81.4%) did not believe that neurological damage was common in children with febrile seizures. Only 27.9% of nurses correctly agreed that alteration of brain metabolism as a result of infection can lower the seizure threshold in children, while 57.1% were unsure. A total of 63.8% of the nurses correctly agreed that the first febrile seizure cannot be prevented, however almost half (47.9%) believed that it was important to treat the fever aggressively with antipyretics to prevent febrile seizures.	The low level of knowledge and inappropriate attitudes of nurses regarding fever and its control result in inconsistent practices that are not always based on up-to-date evidence. If nurses are educating student nurses and newly qualified nurses in the management of fever, the risk of incorrect knowledge being transferred, and inappropriate attitudes being reinforced is high. Nurses who may have irrational fears about fever educate and counsel parents of children with fever, there is a risk that nurses will promote, rather than alleviate, fever phobia in parents	MEDIUM	3
Walsh et al. 2005	Descriptive	Questionnaire	Describe the knowledge and attitudes of Australian paediatric nurses regarding fever and its management, as well as the prognosis of their intentions to administer paracetamol to a febrile child.	Australia. Does not specify date of completion.	**Población**: Level 1 and 2 nurses on the floors of a metropolitan paediatric hospital. **Sample**: 51 paediatric nurses.	62% of the knowledge items were answered correctly. Nurses reported positive attitudes towards the benefits of fever (68%) and that external cooling methods can cause chills, an undesirable fact (88%). Inappropriate attitudes were reflected in disbelief that childhood temperatures are often unrelated to disease severity (52%). Most participants agreed that regular administration of antipyretics could mask fever indicative of an infectious process (94%). Some believed that paracetamol was necessary for all children with temperatures of 38 °C or higher (31.4%), and that sleeping children with temperatures of 38 °C or higher needed to be awakened for an antipyretic (37.3%), and that temperature was the basis for administration of an antipyretic (39.2%). The nurses correctly believed that initial febrile seizures cannot be prevented (90.2%) and that they do not cause neurological damage (92.1%). Many believed that it was necessary to prevent febrile seizures in all children by treating the fever aggressively with antipyretics (86.2%).	The current practices of the nurses in this study were inappropriate, as they advocated the use of antipyretics to prevent febrile seizures and the reduction of low temperatures such as 38 °C. Fever generation is protective; pharmacological efforts to reduce it can be harmful. Fever control should always be based on a thorough understanding of the febrile response and on a thorough individual assessment and response to fever.	MEDIUM	3
Edwards et al. 2003	Descriptive- observational	Audit	Document the practices of nurses in relation to the administration antipyretics by Pro Re Nata order “prn” by doctors for children with fever	Australia. March 2000.	**Population**: Level 2 nurses from a third level paediatric hospital. Data on administration of antipyretics to children with fever who were admitted for 7 months. **Sample**: Data on 67 children from 3 months to 10 years.	The administration of antipyretics generally followed the average temperatures, except at 0800 h and 1600 h, which coincide with the start of new shifts. 51 children (76.1%) received at least one dose of antipyretics. The average temperature recorded for children receiving an antipyretic was 37.44 °C (SD 1.08).	Febrile children admitted to medical wards are likely to be under 2 years old and unable to communicate their needs to the nurses who care for them. These nurses are responsible for monitoring their condition and controlling their fever. One of the ritual nursing actions associated with fever management is the administration of antipyretics to reduce fever and prevent febrile seizures. Antipyretics do not prevent febrile seizures but interfere with the body’s defence mechanisms to fight the disease. Nurses who practice in this way may be harming the children in their care. This audit of nursing practices has highlighted a deficit in nurses’ documentation practices and a lack of clarity in ordering medicines that have dual action, i.e., antipyretics and analgesics.	MEDIUM	3
**Edwards et al. 2001**	Qualitative	Focal groups	Identify nurses’ practices and decision-making criteria for fever control in children hospitalized with a febrile illness. Explain differences in the administration of antipyretics according to time of day and identify ways to improve nurse management of fever and recording of fever management.	Australia. Does not specify date of completion.	**Population**: All level 1 and 2 nurses working on the two paediatric floors of a metropolitan paediatric teaching hospital. **Sample**: 15 nurses.	For nurses there was no “fixed” temperature, they assess the child and if the child is feverish, but otherwise happy they do not administer an antipyretic. However, for most nurses there is a temperature above which they would administer an antipyretic, regardless of the child’s behaviour. This temperature ranges from 37.5 °C to 39.0 °C, which highlights the differences observed in practice. Some recommended warm fans and sponges. However, others argued against their effectiveness citing recent research. Parental requests are considered in the use of non-pharmacological measures. Nurses comply with the parents’ request and administer an antipyretic. This can create conflicts and difficulties for other nurses working in the area who prefer to observe the child and adds another dimension to decision-making. Fever control practices are often changed for children with a history of febrile seizures. Some nurses administered paracetamol whenever these children were febrile, preferring to keep their temperature low and seeking the advice of the parents to help reduce the child’s temperature. These nurses are concerned about febrile seizures and the effect they have on parents.	Not specified.		Gálvez Toro (Nivel 4)

**Table 2:** Quality of studies measured by “Critical Appraisal Skills Programme España” (CASPe) *. Levels of evidence used: “Scottish Intercollegiate Guidelines Network” SIGN (for studies with quantitative methodology) **; Gálvez Toro method (for studies with qualitative methodology) ***.

First Author & Year	Design	Intervention and Comparison	Objectives	location and Date of Execution	Population	No. of Participants/Group	Measuring Instrument	Analysed Variables	Results	Conclusions	Quality of the Study CASPE *	Levels of Evidence (SIGN **) (Gálvez Toro ***)
Jeong & Kim. 2014	Quasi-experimental with control and intervention groups both with pre-test and post-test	**Intervention**: “Blended learning programme”, mixed programme face-to-face and online **Comparison**: Face to face learning	Compare learning from a blended learning programme on fever management for paediatric nurses with a face-to-face one.	Korea. April to May 2012.	Paediatric nurses from two children’s hospitals.	**Experimental group**: 30 nurses. **Control group**: 29 nurses.	Structured self-administered questionnaire. Adapted from the “Fever Management Survey”, (Walsh et al., 2005): Knowledge: 20 multiple choice questions about awareness of childhood fever and fever management Attitudes: 5-point Likert scale (33 items) Fever (10 items) Fever management (13 items) Febrile convulsion (10 items) Normative Influences: 7-point Likert scale (3 items) Parents Nursing colleagues Medical staff Perceptions of control: 7-point Likert scale (4 items) Intention to use antipyretic drugs: 7-point Likert scale (2 items). ‘I intend to administer antipyretics, when caring for a febrile child in future,’ ‘If antipyretics are ordered, I intend to administer them when caring for a febrile child in future.	**Knowledge**: Knowledge Attitudes: **Attitudes** Intentions to use antipyretics **Perceptions**: Normative influences Perceptions of control.	In both groups, post-test results indicated that knowledge of fever and its management; attitude towards fever management; and intentions to use antipyretics showed statistically significant positive changes when compared to pre-test scores. However, post-test results for both groups indicated that normative influences and control perceptions of fever management had changed slightly and were not statistically significant.	A blended learning programme for paediatric fever management was as effective as a traditional face-to-face learning programme in improving knowledge of paediatric fever management, attitudes and reducing intentions to use antipyretics. Satisfaction with the blended learning programme was greater than the face-to-face learning programme. Although the development of the e-learning programme is costly, it is cost-effective considering its effectiveness in terms of time, space, scheduling requirements and study pace. Therefore, a blended learning programme for paediatric fever management could be a useful and flexible learning method for paediatric nurses.	MEDIUM	**2-**
Baran & Turan, 2018	Quasi-experimental with a single group pre-test and post-test.	**Intervention**: They were given a “training booklet”. **Comparison**: Not applicable.	To investigate the effect on training in the management of fever and febrile seizures given to paediatric nurses.	Turkey. January and July 2016.	Paediatric nurses at a children’s hospital.	**Experimental**: 126 nurses participated in the pre-test and post-test. **Control group**: Not applicable	Structured self-administered questionnaire. 40 True-false type questions regarding convulsion and fever management.	**Knowledge**	The findings show that the level of knowledge before training was 32,000 ± 3,779, while the level of knowledge after training was 35,396 ± 2,109. This increase in the level of knowledge is slight but statistically significant	It was noted that training in the management of febrile seizures and fever received by the nurses increased their level of knowledge.	LOW	**2-**
Edwards et al., 2007	Quasi-experimental, with control and intervention group, with pre-test, post-test and latency-test	**Intervention**: Peer-to-peer education. **Comparison**: No programme is offered to them.	To examine the effectiveness of a theory-based educational programme (peer education) in reducing the inappropriate use of antipyretics in the treatment of fever.	Australia. August to April 2003	Nurses from two hospitals.	**Experimental**: 38 nurses attended session 1, 34 nurses attended session 2, 26 nurses attended session 3 and 20 nurses attended session 4. **Control group:** Not specified	Structured self-administered questionnaire. From “Fever management survey (FMS)” (fever management survey) (Walsh, 2005) Fever management practices (FMP): 28 Questions true-false type Attitudes about the effectiveness of antipyretics (18 items) Normative influences (6 items) Perception of control (2 items)	**Attitudes**: Attitudes about the effectiveness of antipyretics **Perceptions**: Normative influences Perception of control	Attitudes towards the effectiveness of antipyretics in increasing comfort, activity, appetite, alertness and in reducing irritability, risk of fever, parental anxiety and temperature were not significantly influenced by the peer education. Peer education reduced the normative influences of peers, parents, and doctors on the administration of antipyretics by nurses to febrile children. The nurses in the experimental group were more aware of the factors that exerted a controlling influence on their administration of antipyretics to febrile children than the nurses in the control group and their intention to administer antipyretics was reduced.	The treatment of fever by paediatric nurses before the peer education was inconsistent and ritualistic. Afterwards, the practices of nurses working on specific wards in the experimental hospital were in line with the latest scientific evidence. Education precipitated change in the factors influencing fever management by paediatric nurses, specifically normative influences, perceptions of control, and intentions to administer antipyretics to febrile children. Nurses became aware of their perceived normative beliefs about the administration of antipyretics and the influence these have on their practice.	MEDIUM	**2-**
Considine & Brennan, 2006	Quasi-experimental with a single pre and post-test group	**Intervention**: Effect of an evidence-based educational programme intended to change the views of emergency nurses on paediatric fever. **Comparison**: Not applicable	To study the opinions of emergency nurses on paediatric fever, and the effect of an evidence-based educational programme to change it	Australia. 2005.	Paediatric emergency nurses.	**Experimental**: 52 nurses. **Control group**: Not applicable	Structured self-administered questionnaire. The ‘General Opinions about Fever Management in Children’ sur-vey 5-point Likert scale (33 items) (Walsh, 2005) Fever 10 items Antipyretics 13 items Febrile convulsion 10 items	**Attitudes**: Fever Antipyretics Febrile convulsion	The number of nurses who agreed that temperature in children is not usually related to disease severity increased by 22.6% (*p* = 0.020) and there was a 35.5% increase in the number of nurses who agreed that temperatures below 41 °C may not be harmful (*p* = 0.001). Although 100% of participants agreed that parents have a phobia of fever in both the pre- and post-test, the number of nurses who agreed that nurses also share this phobia increased by 38.7% (*p* = 0.003). The number of nurses who agreed that nurses should determine when paracetamol should be given increased by 35.5% (*p* = 0.003). There was a 29.1% decrease in the number of nurses who agreed that it is important to aggressively treat fever in children with a history of febrile seizures.	Emergency nurses are an important source of information for parents leaving emergency department care with a feverish child. Opinions can be an important influence on nurses’ clinical decisions and many of the fever management strategies used by health professionals reflect individual beliefs rather than the best available evidence. The results of this study showed several positive changes in the opinions of emergency nurses regarding paediatric fever.	LOW	**2-**
Considine & Brennan, 2007	Quasi-experimental with a single pre and post-test group	**Intervention**: Evidence-based educational intervention about the treatment of paediatric fever **Comparison**: Not applicable	Examine emergency nurses on frequency and autonomy of decisions on treatment of paediatric fever, factual knowledge on treatment of paediatric fever, and knowledge acquisition following an evidence-based educational intervention on treatment of paediatric fever.	Australia. June to August 2005.	Paediatric emergency nurses.	**Experimental**: 52 nurses **Control group**: Not applicable	Structured self-administered questionnaire. 20 multiple choice questions about fever management	**Knowledge**	Out of 15 multiple choice questions (MCQ) on average, they answered 3.62 questions more correctly in the post-test than in the pre-test. The average increase in knowledge after education was 3.62 MCQ (SD = 2.31).	Emergency nurses play an important role in the treatment of febrile children. Factual knowledge about the treatment of paediatric fever has increased with education, and it can be assumed that educational interventions that increase knowledge will improve clinical decision-making. The high levels of variability in knowledge and knowledge acquisition suggest that a review of curricula is warranted.	LOW	**2-**
Edwards et al., 2007	Quasi-experimental with control and intervention group	**Intervention**: Peer-to-peer education **Comparison**: They continue their regular practice	Evaluation of the effectiveness of a peer education programme to develop evidence-based knowledge and attitudes of paediatric nurses for the treatment of fever and the sustainability of such changes	Australia. August 2002 to March 2003.	Level 1 and Level 2 nurses from two Australian hospitals	Does not clearly specify the number of participants per group (77 nurses from the two hospitals)	Structured self-administered questionnaire. From “Fever management survey (FMS)” (fever management survey) (Walsh, 2005) Fever management knowledge (FMK) (24 multiple choice questions) The physiology of fever (10 items) General fever management principles (7 items) Antipyretic medications (7 items) Fever management attitudes (FMA) (32 items, 5-point Likert scale) Fever (9 items) Fever management (14 items) Febrile convulsion (7 items) Parent education (2 items)	**Knowledge**: Physiology of fever Fever management Antipyretics **Attitudes**: Attitude towards evidence-based fever management	Nurses in the experimental groups reported significantly greater overall knowledge when latency data were collected compared to pre-test data. No significant differences in knowledge of antipyretics were found between or within groups at the three data collection time points. Examination of the main single effects revealed reports of significantly more positive attitudes towards evidence-based fever management by the experimental group than the nurses in the control group in the post-test data and latency than in the pre-test.	Fever management is a universal fact of life for nurses caring for children, regardless of environment or country. The identification of current knowledge and attitudes and their influence on practice highlighted the need for change among those who attended the peer education (PEP). The long-term effectiveness of PEP and its adaptability to other environments and cultures needs to be assessed.	LOW	**2-**

## Data Availability

The data of this study are the included tables, figures, and referenced articles.
